# Effects of non-HDL-C and statin therapy on mortality in ARDS: a retrospective cohort study

**DOI:** 10.3389/fmed.2025.1594164

**Published:** 2025-07-30

**Authors:** Haiming Hu, Jin Wei, Lijuan Zhao, Yixing Zhu, Geer Zhou, Anqi Zhao, De Chang

**Affiliations:** ^1^Department of Pulmonary and Critical Care Medicine at The Seventh Medical Center, Beijing, China; ^2^College of Pulmonary and Critical Care Medicine of The Eighth Medical Center, Chinese PLA General Hospital, Beijing, China; ^3^Graduate School of Chinese PLA General Hospital, Beijing, China; ^4^Department of Respiratory and Critical Care Medicine, The Second Affiliated Hospital of Air Force Medical University, Xi’an, China; ^5^Department of Medical Oncology, Senior Department of Oncology, The Fifth Medical Center of PLA General Hospital, Beijing, China

**Keywords:** ARDS, statin, non-HDL-C, clinical prognosis, MIMIC-IV database

## Abstract

**Background:**

Acute respiratory distress syndrome (ARDS) is a critical and potentially fatal condition marked by inflammation and coagulation disorders. Statins, a class of cholesterol-lowering medications, have been explored for potential anti-inflammatory properties, yet their exact role in ARDS remains unclear.

**Methods:**

Patients diagnosed with ARDS were sourced from the MIMIC-IV database (version 3.0). To balance baseline characteristics, propensity score matching (PSM) was applied. Short-term mortality was evaluated using Kaplan–Meier survival analysis. Factors associated with short-term mortality were determined using both univariate and multivariate Cox regression analyses. The potential impact of unmeasured confounding was assessed using the E-value. Additionally, subgroup analyses were performed to investigate heterogeneity and evaluate the robustness of the findings.

**Results:**

The study included 10,368 ARDS patients, of whom 5,184 received statin therapy and 5,184 did not. Kaplan–Meier analysis revealed significantly lower short-term mortality in the statin-treated group. Both univariate (HR, 0.48; 95% CI, 0.41–0.58; *p* < 0.001) and multivariate (HR, 0.49; 95% CI, 0.41–0.58; *p* < 0.001) Cox regression analyses revealed that statin therapy significantly decreased short-term mortality. Subsequent subgroup analyses further indicated that the beneficial effect of statins was more evident in patients with elevated non-HDL-C levels.

**Conclusion:**

Statin therapy appears to confer significant clinical benefits in ARDS patients, particularly in those with high non-HDL-C levels. These findings indicate that non-HDL-C might be a useful marker for identifying ARDS patients who may benefit most from statin therapy.

## Introduction

Acute respiratory distress syndrome (ARDS) represents a critical and advancing clinical scenario marked by persistent hypoxemia (1). Initially described by Ashbaugh et al. (2), ARDS stands as a major contributor to acute respiratory failure, which is linked to considerable illness and death rates, especially among individuals with underlying health conditions (3). The prevalence of ARDS among intensive care unit (ICU) patients is estimated to be approximately 10.4%, with mortality rates exceeding 35% (4). Despite advancements in critical care management, effective therapeutic interventions remain elusive (5). The current understanding of ARDS pathophysiology emphasizes the activation and dysregulation of intertwined inflammatory and coagulation pathways. A revised definition of ARDS, which incorporates oxygen saturation as a marker of hypoxemia, offers a more practical and clinically relevant framework for assessing acute hypoxic respiratory failure, particularly in the early stages of the syndrome (6). Furthermore, the identification of distinct inflammatory phenotypes of ARDS has the potential to refine therapeutic strategies, facilitating more targeted interventions (7, 8). Consequently, the use of tailored anti-inflammatory therapies is increasingly advocated to improve patient outcomes (9).

Statins, which are inhibitors of hydroxymethyl-glutaryl-coenzyme A (HMG-CoA) reductase, have shown potential in reducing inflammatory responses and mitigating acute lung injury (10, 11). Previous studies have suggested that statins can reduce inflammation and improve clinical outcomes in a variety of conditions. A meta-analysis revealed that the use of statins was significantly linked to a reduced mortality rate (relative risk [RR], 0.65; 95% confidence interval [CI], 0.57–0.75) in sepsis patients (12). However, two large RCTs, the HARP-2 trial (focusing on simvastatin) and the SAILS study (assessing rosuvastatin in ARDS patients), did not show an overall benefit in ARDS outcomes. Furthermore, the use of statins was linked to an increased incidence of non-serious adverse events (13, 14). Notably, secondary analyses of these studies suggested that patient subgroups, defined by specific phenotypic characteristics such as variations in inflammatory markers or cholesterol levels, may experience divergent treatment outcomes (8, 15). For example, simvastatin has demonstrated cost-effectiveness and a notable improvement in quality-adjusted life years (QALYs) (16).

In this retrospective analysis, we examined the impact of statin treatment on short-term mortality among ARDS patients. Additionally, we conducted subgroup analyses to identify the conditions that maximize the clinical advantages of statins. Our analysis was based on the updated 2024 ARDS criteria and data from a publicly available clinical database to inform our analysis.

## Methods

### Data sources

This retrospective study was conducted using patient records from the MIMIC-IV (version 3.0) (17). Comprising detailed healthcare data, the MIMIC-IV dataset covers 546,028 admissions to critical care units at Beth Israel Deaconess Medical Center (18). Upon successfully completing the required training and the examination stipulated by the NIH, author Haiming Hu was granted access to the database, enabling the extraction of the essential data.

### Population selection

**Figure 1:**
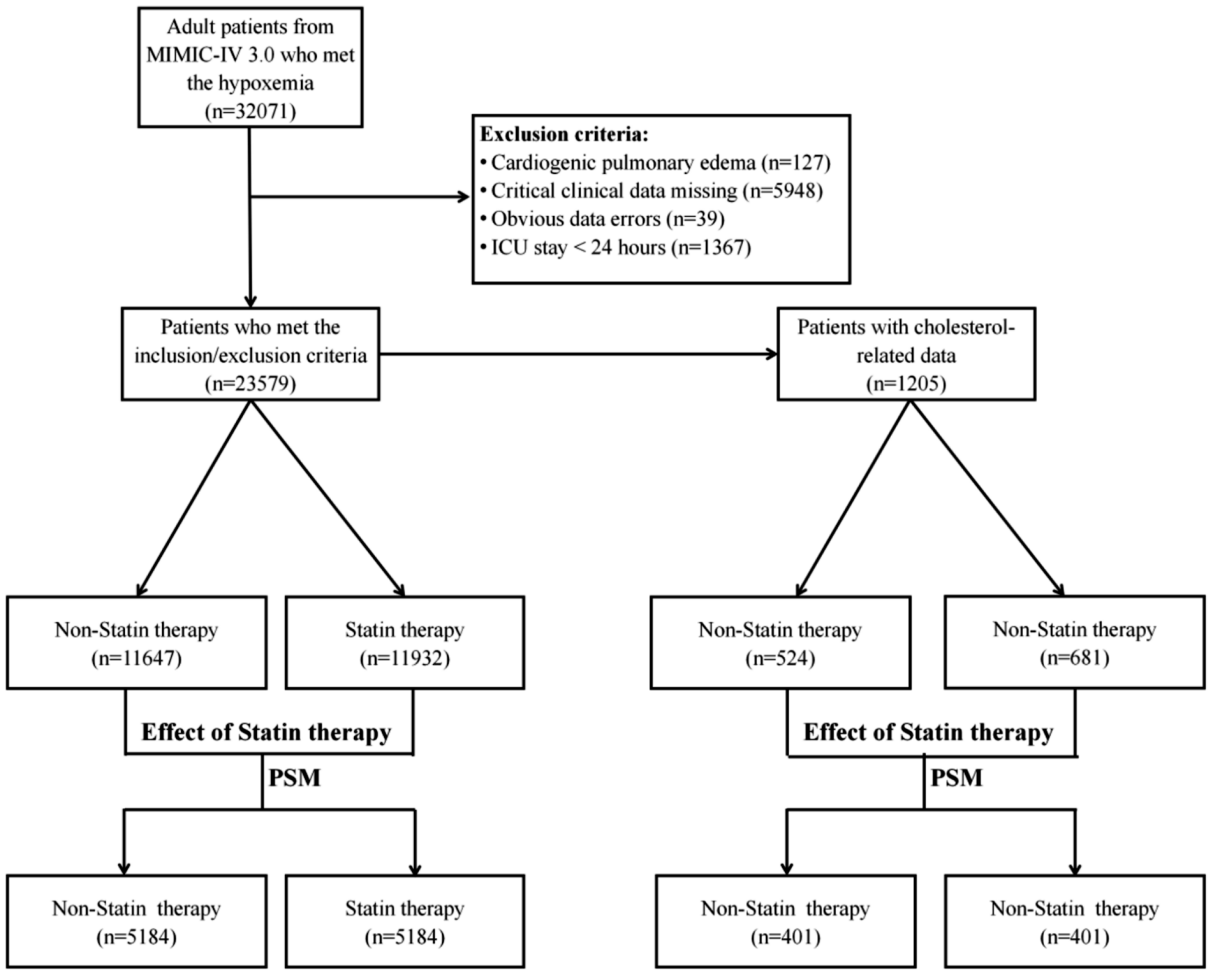
Flowchart illustrating the patient selection process for the study analysis.

This study comprised adult patients with a diagnosis of ARDS in ICUs, according to the Berlin definition or the new global definition (6, 19). The inclusion criteria were based on PaO2/FiO2 ≤ 300 mmHg with PEEP or CPAP ≥ 5 cm H2O, or SPO2/FiO2 ≤ 315 when pulse oximetry saturation was ≤ 97%. The following criteria were used for exclusion: (1) age < 18 year; (2) cardiogenic pulmonary edema; (3) critical clinical data missing, including blood-gas analyses and vital signs; (4) apparent errors in clinical data. Statin therapy was defined as the administration of statins to patients for treatment purposes. To ensure data consistency, we consolidated multiple ICU visits by the same patient within one hospital admission into one record. Figure 1 illustrates the flowchart.

### Data acquisition and definition criteria

Data retrieval from the MIMIC-IV database was executed utilizing SQL through Navicat Premium software, version 13.3.2. Comorbidities were determined based on documented diagnoses. Specifically, liver-related conditions encompassed hypohepatia, liver failure, and other associated pathologies. Regarding renal comorbidities, they comprised chronic renal insufficiency, uremia, and other relevant disorders. Tumors included liver cancer, lung cancer, and other related conditions. Statin therapy included atorvastatin, simvastatin, rosuvastatin, and other related agents. Glucocorticoid therapy involved the administration of methylprednisolone, dexamethasone, and other equivalent corticosteroids. Patients were categorized as survivors if they were discharged prior to the observation endpoint. Only those who died during their hospital stay were classified as deceased. Additionally, patients who died were excluded from the calculation of length of ICU and hospital stay.

### Clinical endpoints

The primary endpoint was short-term mortality (7-day mortality). The secondary endpoints encompassed 30-day, 90-day, the length of ICU and overall hospital stays, rate of mechanical ventilation, and the incidence of liver and renal disease.

### Statistical analysis

In this study, individuals were divided into two groups based on statin treatment status. For normally distributed continuous variables, we present the mean with standard deviation (SD), whereas for those not normally distributed, we provide the median and interquartile range (IQR). Categorical variables are expressed in terms of counts and percentages. To evaluate the differences in baseline characteristics between groups, we applied the t-test or Mann–Whitney U test to continuous variables, contingent upon their distribution. The Pearson Chi-Square (χ^2^) test was utilized for categorical variables to determine statistical significance.

Propensity score matching (PSM) was used with a 0.01 caliper to reduce confounding and baseline differences. The cohort underwent 1:1 matching via nearest neighbor technique. The nuclear density map before and after PSM is shown in Supplementary Figure S1. To begin with, a univariate Cox regression analysis was executed to pinpoint variables linked to patient outcomes, discarding those without statistical relevance. Subsequently, the selected variables were integrated into a stepwise Cox regression model, which was then developed into a comprehensive multivariate Cox model for the purpose of assessing short-term mortality rates among patients with ARDS. The E-value, which stems from the correlation observed between statin treatment and mortality rates, signifies the minimum strength of relationship that an unaccounted confounder must have with both the treatment and the mortality outcome. This is based on the covariates that have been measured, to fully explain the observed link between the treatment and the mortality outcome (20, 21).

Survival analysis was used to depict the survival probabilities associated with statin therapy, and log-rank test was utilized to assess the statistical significance of differences. Additionally, the associations between mortality risk and several laboratory parameters were examined using four-knot RCS models within the framework of Cox proportional hazards regression. Subgroup analyses were conducted to investigate the most effective timing for statin administration, categorized by factors such as age (≤ 65 years and > 65 years), WBC counts (≤ 7.5 × 10^9/L and >7.5 × 10^9/L), hemoglobin (≤ 10.5 g/dL and > 10.5 g/dL), INR (≤ 1.3 and > 1.3), disease severity (mild: SpO2/FiO2 > 235; moderate: 148 < SpO2/FiO2 ≤ 235; severe: SpO2/FiO2 ≤ 148), CCI (≤ 6 and >6), SAPS II score (≤ 40 and > 40), SOFA score (< 2 and ≥ 2) and comorbidities. Additionally, various therapeutic approaches were considered, including glucocorticoid and aspirin. The variance-ratio test was applied to assess subgroup interactions. Moreover, based on the results of the RCS curve analysis and the specific features of the dataset, WBC counts, hemoglobin levels, and INR were stratified into distinct subgroups.

All statistical tests were executed with R software, version 4.4.1. A threshold of *p* < 0.05, two-tailed, was set for statistical significance. Results are presented in terms of hazard ratios (HRs) along with their respective 95%CIs.

## Results

### Baseline characteristics

This study enrolled 23,579 ARDS patients who met the inclusion and exclusion criteria. In the original cohort, the statin group exhibited a higher mean age and a lower SpO₂/FiO₂ ratio compared to the non-statin group. Following PSM, 5,184 matched pairs were identified. Among the enrolled patients, 4,269 (41.2%) were female and 6,099 (58.8%) were male. The average age of the matched cohort was 69 years (SD, 13). The statin group demonstrated a lower SpO₂/FiO₂ ratio and a higher incidence of myocardial infarction. Table 1 presents a comprehensive comparison of the clinical characteristics.

**Table 1:** Demographic and clinical characteristics of ARDS patients with and without statin treatment before and after PSM.

Character	Original cohort		*p*-value	Matched cohort		*p*-value
	Non-statin	Statin		Non-statin	Statin	
Patients	*N* = 11,647	*N* = 11,932		*N* = 5,184	*N* = 5,184	
Age [years, M (SD)]	61 (17)	71 (12)	<0.001	69 (14)	69 (12)	0.031
Sex			<0.001			0.968
Male [*n* (%)]	6,590 (56.6)	7,622 (63.9)		3,051 (58.9)	3,048 (58.8)	
Female [*n* (%)]	5,057 (43.4)	4,310 (36.1)		2,133 (41.1)	2,136 (41.2)	
Vital signs						
Heart rate [/min, M (SD)]	121 (23)	114 (24)	<0.001	117 (23)	117 (25)	0.586
Temperature [°C, M (SD)]	38.0 (0.9)	37.8 (0.8)	<0.001	37.9 (0.8)	37.9 (0.8)	0.888
SBP [mmHg, M (SD)]	164 (27)	161 (26)	<0.001	164 (27)	164 (27)	0.975
DBP [mmHg, M (SD)]	41 (11)	39 (10)	<0.001	40 (11)	40 (10)	0.532
RR [/min, M (SD)]	35 (9)	33 (8)	<0.001	34 (8)	34 (8)	0.891
Spo2 [%, M (SD)]	87 (8)	88 (7)	<0.001	87 (7)	87 (7)	0.248
Spo2/FiO2 [M (SD)]	198 (51)	194 (46)	<0.001	198 (49)	195 (48)	<0.001
Laboratory data						
WBC [× 10^9/L, M (SD)]	12.5 (10.2)	11.6 (8.0)	<0.001	12.1 (9.1)	12.0 (9.3)	0.714
Platelet [× 10^9/L, M (SD)]	211.6 (123.0)	212.0 (99.2)	0.777	214.1 (120.0)	215.9 (104.4)	0.414
Hemoglobin [g/L, M (SD)]	10.9 (2.4)	11.0 (2.4)	0.007	10.9 (2.3)	10.9 (2.4)	0.671
INR [M (SD)]	1.5 (0.9)	1.5 (0.8)	<0.001	1.5 (0.9)	1.5 (0.9)	0.958
Clinically scores						
CCI [M (SD)]	5 (3)	7 (3)	<0.001	6 (3)	6 (3)	0.945
SAPS II scores [M (SD)]	39 (15)	40 (13)	<0.001	41 (14)	40 (14)	0.296
SOFA scores [M (SD)]	2 (3)	2 (2)	0.002	2 (2)	2 (2)	0.958
Comorbidity						
Hypertension [*n* (%)]	5,404 (46.4)	6,292 (52.7)	<0.001	2,760 (53.2)	2,728 (52.6)	0.542
Asthma [*n* (%)]	1,013 (8.7)	949 (8.0)	0.041	435 (8.4)	420 (8.1)	0.617
COPD [*n* (%)]	703 (6.0)	1,203 (10.1)	<0.001	459 (8.9)	461 (8.9)	0.972
Diabetes [*n* (%)]	2,673 (23.0)	5,269 (44.2)	<0.001	1734 (33.4)	1819 (35.1)	0.082
Heart failure [*n* (%)]	2,418 (20.8)	4,899 (41.1)	<0.001	1,669 (32.2)	1702 (32.8)	0.502
Myocardial infarction [*n* (%)]	688 (5.9)	3,205 (26.9)	<0.001	592 (11.4)	667 (12.9)	0.026
Stroke [*n* (%)]	21 (0.2)	34 (0.3)	0.126	14 (0.3)	12 (0.2)	0.844
Pulmonary embolism [*n* (%)]	835 (7.2)	549 (4.6)	<0.001	330 (6.4)	328 (6.3)	0.968
Venous thrombosis [*n* (%)]	690 (5.9)	553 (4.6)	<0.001	305 (5.9)	289 (5.6)	0.526
Tumor [*n* (%)]	102 (0.9)	58 (0.5)	<0.001	30 (0.6)	32 (0.6)	0.899
Treatment						
Glucocorticoid use [*n* (%)]	4,022 (34.5)	2,618 (21.9)	<0.001	1,484 (28.6)	1,486 (28.7)	0.983
Aspirin therapy [*n* (%)]	3,319 (28.5)	9,636 (80.8)	<0.001	3,015 (58.2)	3,011 (58.1)	0.952

Continuous variables are depicted as medians with interquartile ranges (IQRs) for data that do not follow a normal distribution. Categorical variables are represented through counts and corresponding percentages (%).

### Impact of statin therapy on clinical outcomes

**Figure 2:**
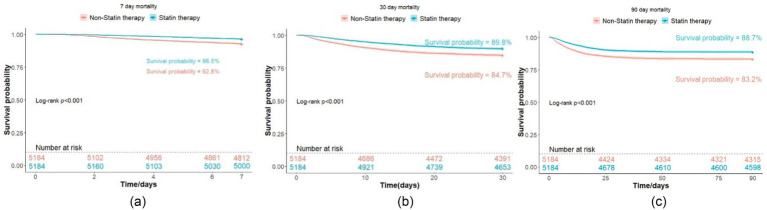
K-M survival curves for ARDS patient mortality. **(a)** 7-day mortality; **(b)** 30-day mortality; **(c)** 90-day mortality.

Statin therapy was linked to significantly lower rates of short-term mortality (3.5% vs. 7.2%, *p* < 0.001), as well as 30-day mortality (10.2% vs. 15.3%, *p* < 0.001), 90-day mortality (11.3% vs. 16.8%, *p* < 0.001), and in-hospital mortality (12.3% vs. 17.8%, *p* < 0.001) compared to non-users. The Kaplan–Meier survival analysis showed significant improvements in mortality at all evaluated time points (log-rank test, *p* < 0.001), as illustrated in Figure 2. There was no statistical significance in the comparison of mechanical ventilation rates (68.5% vs. 69.4%, *p* = 0.319), ICU length of stay (mean, 153.1 h vs. 152.2 h, *p* = 0.823), or hospital stay duration (mean, 14.8 days vs. 15.1 days, *p* = 0.354). Nevertheless, statin therapy correlated with a higher incidence of renal disease (6.9% vs. 4.1%, *p* < 0.001) and elevated creatinine levels (1.6 mg/dL vs. 1.4 mg/dL, *p* < 0.001), suggesting a potential association with renal injury. In contrast, statin therapy was linked to a reduced incidence of liver diseases (1.0% vs. 1.6%, *p* = 0.012), suggesting a protective effect against hepatic complications in ARDS patients (22). A summary of these results can be found in Table 2.

**Table 2:** Association of statin treatment and clinical outcomes.

Outcomes	Non-statin	Statin	*p*-value
In-hospital mortality [*n* (%)]	925 (17.8)	639 (12.3)	<0.001
Mortality at day7 [*n* (%)]	372 (7.2)	184 (3.5)	<0.001
Mortality at day30 [*n* (%)]	793 (15.3)	531 (10.2)	<0.001
Mortality at day90 [*n* (%)]	869 (16.8)	586 (11.3)	<0.001
Mechanical ventilation [*n* (%)]	3,598 (69.4)	3,550 (68.5)	0.319
Liver disease [*n* (%)]	83 (1.6)	53 (1.0)	0.012
Renal disease [*n* (%)]	211 (4.1)	357 (6.9)	<0.001
Creatinine [mg/dL, M (SD)]	1.4 (1.4)	1.6 (1.8)	<0.001
Duration of ICU stay [hours, M (SD)]	152.2 (173.0)	153.1 (199.11)	0.823
Duration of Hospital stay [days, M (SD)]	15.1 (14.6)	14.8 (14.9)	0.354

Continuous variables are depicted as medians with interquartile ranges (IQRs) for data that do not follow a normal distribution. Categorical variables are represented through counts and corresponding percentages (%).

### Analyzing short-term mortality predictors in ARDS patients by cox regression models

The univariate Cox regression analysis identified statin therapy (HR, 0.48; 95% CI, 0.41–0.58; *p* < 0.001) and aspirin therapy (HR, 0.46; 95% CI, 0.39–0.55; *p* < 0.001) were significantly linked to a decrease in short-term mortality rates. Additionally, asthma was identified as a protective factor (HR, 0.63; 95% CI, 0.43–0.91; *p* = 0.013). The variables that were statistically or clinically significant in the univariate analysis were included in a stepwise Cox regression model. This model identified ten key prognostic factors: age, SpO₂/FiO₂ ratio, WBC count, INR, asthma, diabetes, myocardial infarction, stroke, statin therapy, and aspirin therapy (Supplementary Table S1). The multivariate analysis confirmed that an elevated SpO₂/FiO₂ ratio (HR, 0.99; 95% CI, 0.99–0.99; *p* < 0.001), along with statin (HR, 0.49; 95% CI, 0.41–0.58; *p* < 0.001), and aspirin therapy (HR, 0.49; 95% CI, 0.41–0.59; *p* < 0.001) were associated with decreased short-term mortality. Conversely, advanced age (HR, 1.04; 95% CI, 1.03–1.04; *p* < 0.001), higher WBC count (HR, 1.01; 95% CI, 1.01–1.01; *p* < 0.001), elevated INR (HR, 1.15; 95% CI, 1.09–1.22; *p* < 0.001), diabetes (HR, 1.26; 95% CI, 1.06–1.49; *p* = 0.009), myocardial infarction (HR, 2.40; 95% CI, 1.98–2.91; *p* < 0.001), and stroke (HR, 4.82; 95% CI, 1.80–12.91; *p* = 0.002) were linked to increased mortality. These findings align with the univariate analysis, confirming their robustness. The E-value, reflecting the influence of statin therapy on the reduction of short-term mortality rates in ARDS patients, stood at 3.59. Additionally, the E-value associated with the 95%CI was 2.84. Multivariate analysis outcomes are depicted in Supplementary Figure S2, complemented by forest plots.

### The impact of statin therapy on short-term mortality within specific subgroups

**Figure 3:**
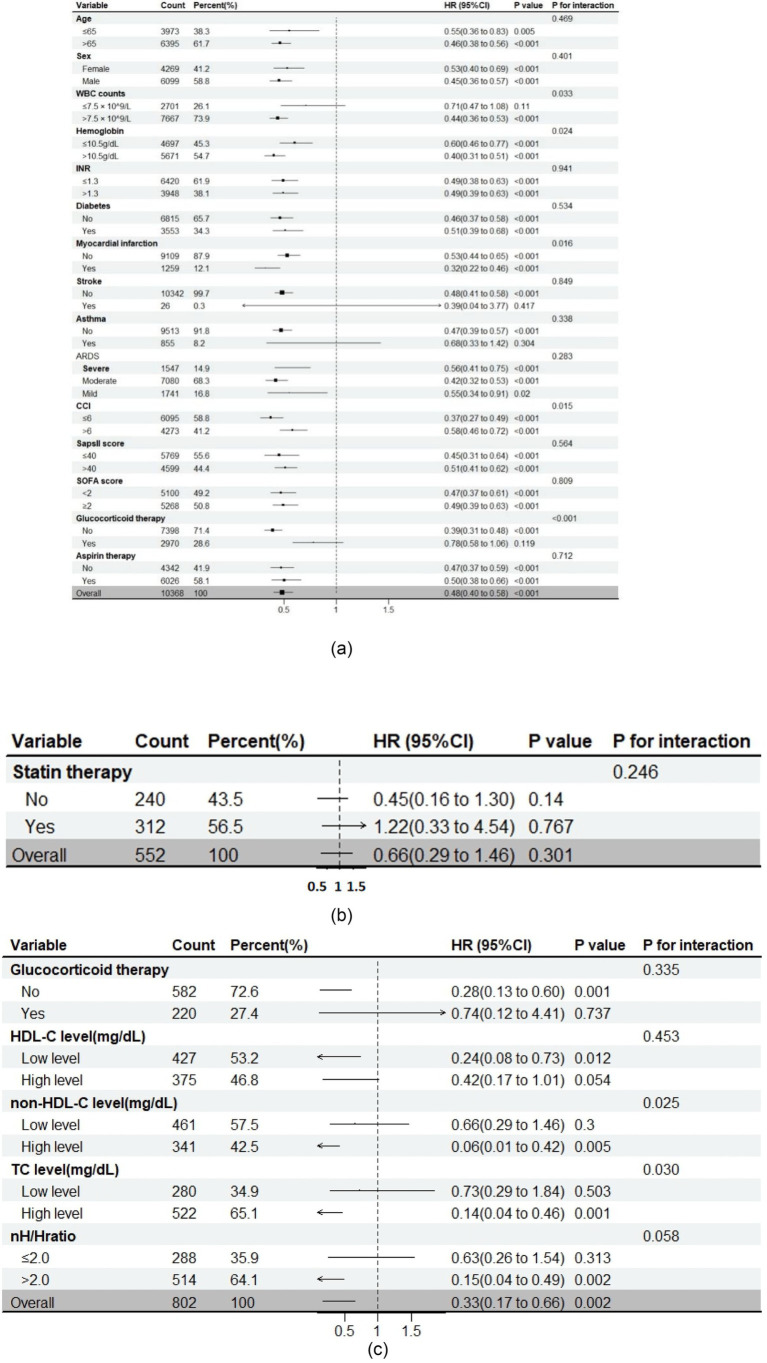
Subgroup analysis on the impact of clinical intervention on 7-day mortality in ARDS patients. **(a)** statin therapy and 7-day mortality (no cholesterol-related data); **(b)** glucocorticoid therapy and 7-day mortality (added cholesterol-related data); **(c)** statin therapy and 7-day mortality (added cholesterol-related data).

To elucidate the association between statin therapy and mortality, subgroup analyses were conducted based on the insights derived from RCS curves and the unique characteristics of data (Supplementary Figure S3). The findings consistently indicated that the use of statins was linked to lower short-term mortality in the majority of subgroups (Figure 3). Significant interaction effects were observed between statin therapy and several factors, including WBC count (*p* = 0.033), hemoglobin (*p* = 0.024), myocardial infarction (*p* = 0.016), CCI (*p* = 0.015), and glucocorticoid therapy (*p* < 0.001). Specifically, statin therapy was more effective in patients with hemoglobin > 10.5 g/dL, WBC count > 7.5 × 10^9/L, CCI > 6, those with prior myocardial infarction, or those not receiving glucocorticoid therapy. No significant interactions were observed with age, sex, INR, diabetes, stroke, ARDS severity, SAPS II score, SOFA score, or aspirin therapy.

### The role of non-HDL-C in ARDS treatment

To explore the diminished effectiveness of statin therapy in ARDS patients undergoing glucocorticoid treatment, we hypothesized that cholesterol variations between subgroups might influence statin effectiveness. We excluded individuals whose records lacked information on total cholesterol (TC), high-density lipoprotein cholesterol (HDL-C), and non-HDL-C (defined as TC minus HDL-C). Baseline comparisons showed that patients receiving glucocorticoid therapy had lower levels of TC (130.0 mg/dL vs. 141.0 mg/dL; *p* = 0.001), HDL-C (34.0 mg/dL vs. 40.0 mg/dL; *p* < 0.001), and non-HDL-C (91.0 mg/dL vs. 99.0 mg/dL; *p* = 0.011) than non-users. Propensity score matching (1:1) corrected for baseline differences (Supplementary Table S2). Subgroup analysis by statin therapy status indicated no significant interaction between glucocorticoid use and statin efficacy, suggesting that cholesterol differences may account for variations in statin response.

From a final cohort of 1,205 patients (524 without statin therapy, 681 with statin therapy), significant differences in TC (134.5 mg/dL vs. 141.0 mg/dL; *p* = 0.003), HDL-C (36.0 mg/dL vs. 39.0 mg/dL; *p* = 0.004), and non-HDL-C (95.0 mg/dL vs. 99.0 mg/dL; *p* = 0.007) were observed between the groups. After 1:1 matching, the baseline characteristics were balanced (Supplementary Table S3). Statin therapy maintained its mortality-reducing effect among ARDS patients (2.7% vs. 8.0%, *p* = 0.002). Subgroup analysis based on cholesterol levels revealed that statins remained effective for patients without glucocorticoid treatment, but showed no significant difference between two group (*p* for interaction = 0.335). Notably, statin therapy demonstrated significant interactions with high levels of TC (*p* = 0.030) and non-HDL-C (*p* = 0.025), and an nH/H ratio > 2.0 (*p* = 0.058). No significant interactions were found with HDL-C. These results suggest that elevated non-HDL-C levels, both in absolute terms and in the nH/H ratio, may be important in optimizing statin therapy for ARDS patients, with potential clinical implications for guiding treatment.

## Discussion

This retrospective study harnessed data from the MIMIC-IV database to examine the influence of statin therapy on ARDS patients. Patients were screened according to the updated definition of ARDS (6), and ultimately included 10,368 patients in this study after applying the inclusion and exclusion criteria and performing PSM.

Our study revealed that statin therapy could reduce the short-term mortality of ARDS patients (3.2% vs. 9.6%, *p* < 0.001). This finding was supported by both univariate (HR, 0.48; 95%CI, 0.41–0.58; *p* < 0.001) and multivariate (HR, 0.49; 95%CI, 0.41–0.58; *p* < 0.001) Cox regression analyses. The E-value of 3.59 was calculated for the observed relationship, implying that it is unlikely that the connection between anticoagulant therapy and mortality is solely due to some unmeasured factor. Statins also contributed to improved 30-day (10.2% vs. 15.3%, *p* < 0.001), 90-day (11.3% vs. 16.8%, *p* < 0.001), and hospital mortality (12.3% vs. 17.8%, *p* < 0.001), with Kaplan–Meier analyses confirming these differences (log-rank test: *p* < 0.001). Nonetheless, there were no notable differences in mechanical ventilation rates (68.5% vs. 69.4%, *p* = 0.319), ICU stays (153.1 vs. 152.2 h, *p* = 0.823), or length of hospital stays (14.8 vs. 15.1 days, *p* = 0.354) between statin users and non-users. Notably, statin therapy was associated with an increased incidence of renal diseases (6.9% vs. 4.1%, *p* < 0.001) and higher average creatinine levels (1.6 mg/dL vs. 1.4 mg/dL, *p* < 0.001), suggesting a potential link between statin use and renal injury. The renally impaired baseline of these patients could potentially make them more susceptible to drug-induced renal injury from drugs like statin (23). However, the exact contribution of drug-induced nephrotoxicity or other confounding factors remains to be determined. Additional studies are necessary to clarify the mechanisms behind this relationship. Conversely, the rate of liver diseases was reduced in the statin group (1.0% vs. 1.6%, *p* = 0.012), suggesting that statins may offer a protective effect for ARDS patients, which aligns with prior studies (24, 25).

Our multivariate Cox regression analysis revealed that higher SpO2/FiO2 (HR, 0.99; 95% CI, 0.99–0.99; *p* < 0.001), asthma (HR, 0.71; 95% CI, 0.49–1.03; *p* = 0.073), statin therapy (HR, 0.49; 95% CI, 0.41–0.58; *p* < 0.001), and aspirin therapy (HR, 0.49; 95% CI, 0.41–0.59; *p* < 0.001) were correlated with lower short-term mortality in ARDS patients. In contrast, increased short-term mortality was associated with several factors: older age (HR, 1.04; 95% CI, 1.03–1.04; *p* < 0.001), higher WBC count (HR, 1.01; 95% CI, 1.01–1.01; *p* < 0.001), elevated INR (HR, 1.15; 95% CI, 1.09–1.22; *p* < 0.001), diabetes (HR, 1.26; 95% CI, 1.06–1.49; *p* = 0.009), myocardial infarction (HR, 2.40; 95% CI, 1.98–2.91; *p* < 0.001), and stroke (HR, 4.82; 95% CI, 1.80–12.91; *p* = 0.002) (26). Aspirin may improve pulmonary tissue damaging in ARDS patients by correcting clotting disorders, suggesting its potential as a potential treatment for ARDS (27, 28). Previous studies indicate that individuals with asthma who contract COVID-19 might have a better prognosis (29, 30), potentially due to increased eosinophil levels or long-term glucocorticoid inhalation. Similarly, we found that asthma was a protective factor for patients with ARDS.

In our initial subgroup analyses, statin therapy correlated with lower short-term mortality in the majority of the subgroups. We observed significant interaction effects between statin use and various factors, including white blood cell (WBC) count (*p* = 0.033), hemoglobin levels (*p* = 0.024), myocardial infarction (*p* = 0.016), CCI score (*p* = 0.015), and glucocorticoid therapy (*p* < 0.001). There were no notable interactions observed among factors including age, sex, INR, diabetes, stroke, severity of ARDS, SAPS II score, SOFA score, and aspirin therapy. The differences in WBC count between the two subgroups may reflect distinct inflammatory phenotypes of ARDS (8, 31). The reduction in statin effectiveness among glucocorticoid users may be due to several factors. Glucocorticoids can affect lipid metabolism, potentially reducing the lipid-lowering impact of statins (32). They also have anti-inflammatory effects, which might interact with statins’ potential anti-inflammatory mechanisms (33). This could alter how patients respond to statin therapy.

To investigate the reasons for the reduced effectiveness of statins in individuals undergoing glucocorticoid treatment, we omitted participants with lacking data on TC, HDL-C, and non-HDL-C from our analysis. We then performed a 1:1 PSM to minimize differences between the two groups. A subgroup analysis based on statin therapy status revealed no significant differences in the effects of glucocorticoid therapy between the subgroups. This suggests that the variations in statin efficacy within the glucocorticoid subgroup may be attributed to differences in cholesterol levels between the two groups. Subsequently, we included 1,205 patients from the original cohort of 10,368, excluding those with missing cholesterol data. After performing 1:1 PSM, the analysis showed balanced baseline characteristics. Upon repeating the subgroup analysis, we found that statins remained effective in the non-glucocorticoid subgroup, even though the difference did not reach statistical significance (*p* = 0.335). However, statin therapy demonstrated significant interaction effects with high levels of TC (*p* = 0.030) and non-HDL-C (*p* for interaction = 0.025), as well as with a ratio of non-HDL-C to HDL-C (nH/H) ratio > 2.0 (*p* = 0.058). No significant interactions were observed with HDL-C levels. Importantly, these results suggest that non-HDL-C, a known predictor of heart disease (34, 35), could have significant clinical relevance in guiding therapeutic approaches for patients with ARDS.

Statins, a category of HMG-CoA reductase inhibitors used for reducing cholesterol, have been found to possess anti-inflammatory effects (36). Theoretically, statins could improve the prognosis of ARDS patients by mitigating inflammatory dysregulation. However, neither the SAILS nor the HARP-2 trials demonstrated benefits of statins in the general ARDS population (13, 14). Nevertheless, secondary analyses suggest that certain subphenotypes, such as those exhibiting hyper-inflammatory characteristics, may respond differently to statin therapy (8). Our analysis supports the potential benefit of statins in ARDS patients and suggests that non-HDL-C could serve as an important clinical marker for guiding statin use. In contrast to previous findings (15), our study identified a subgroup with high total cholesterol in which statin exhibited enhanced efficacy. Additionally, our research indicates that statin could be particularly effective in ARDS patients with elevated non-HDL-C levels, and it is recommended that lipid levels be taken into account in forthcoming trials investigating statin therapy for ARDS patients.

Our research has several advantages. Primarily, our data were sourced from a publicly available database, and the sample size is larger than those in previous studies, ensuring both reliability and comprehensiveness. Second, we utilized the updated definition of ARDS, focusing on its early stages, which provides a more relevant and inclusive approach compared to earlier studies. Third, we employed a combination of univariate, stepwise, and multivariate Cox regression analyses, along with PSM, to enhance the reliability and robustness of our results. To our knowledge, no prior research has explored the role of non-HDL-C in statin-treated ARDS patients. Our findings provide evidence that statin therapy is more effective in subgroups with high non-HDL-C levels, which may help guide clinical decisions regarding statin use in ARDS patients.

This research has several limitations. Principally, because our data are derived exclusively from one database, the lack of external validation may limit the generalizability of our findings to the broader ARDS patient population. Second, we did not conduct further subgroup analyses, such as the effects of statin therapy on ARDS etiology, statin type, drug dosage, and duration of use, which could potentially narrow the applicability of our conclusions. Furthermore, our analysis was constrained by the absence of some laboratory variables, including procalcitonin and interleukin-6 (IL-6), due to gaps in data availability. These biomarkers are crucial for a thorough assessment of the clinical status of ARDS patients. Despite this, we minimized the impact of known confounding variables via PSM. Besides, our sensitivity analysis produced an E-value of 3.59, indicating that the observed correlation between statin treatment and short-term mortality among ARDS patients is robust and not likely confounded by unmeasured factors. Finally, the inherent traits of the database, including a significant number of cases with unspecified ARDS causes in the diagnostic records, coupled with the limitations inherent to a retrospective study design, limited our ability to perform additional subgroup analyses.

## Conclusion

In conclusion, we found that statin therapy showed potential clinical benefits in patients with ARDS. Specifically, statin use significantly reduced the short-term mortality, as well as 30-day and 90-day mortality rates, and also led to a decreased rate of liver disease. Notably, statin therapy demonstrated enhanced efficacy in subgroups with elevated non-HDL-C levels. These findings imply that statins may be a valuable therapeutic strategy for ARDS patients, particularly those with high non-HDL-C levels, warranting further investigation into their potential as a targeted treatment option.

## Data Availability

The original contributions presented in the study are included in the article/Supplementary material, further inquiries can be directed to the corresponding author.
