# Supplementary material for: Effects of non-HDL-C and statin therapy on mortality in ARDS: a retrospective cohort study
Source: Front Med (Lausanne). 2025 Jul 30;12:1594164. doi: 10.3389/fmed.2025.1594164 (PMC12345366; doi:10.3389/fmed.2025.1594164)
Supplement: Supplementary file 1 [file Table_1.DOCX]

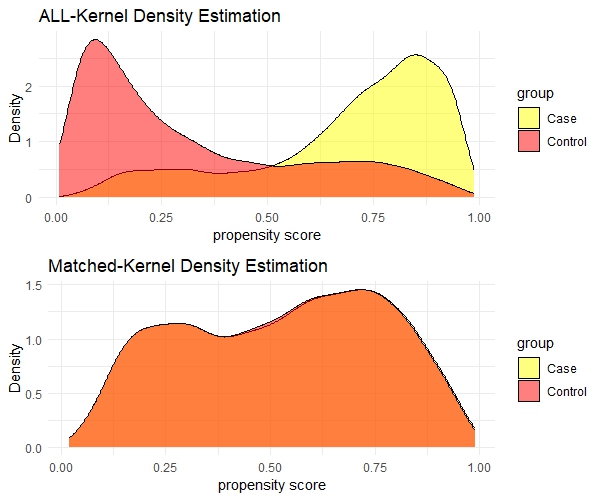


**Figure S1** The nuclear density maps before and after PSM matching. PSM: propensity score matching


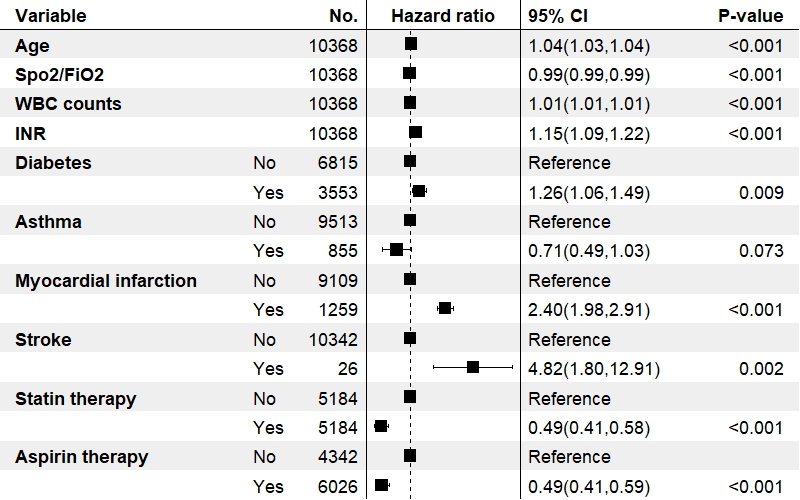


**Figure S2** Multivariate analysis with Cox regression for short-term mortality and corresponding forest plot. WBC: white blood cell count; INR: international normalized ratio


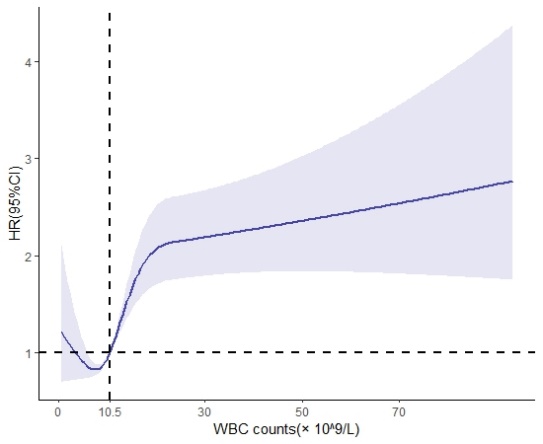

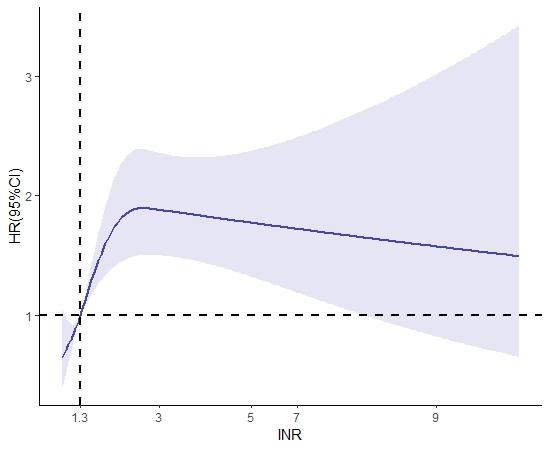

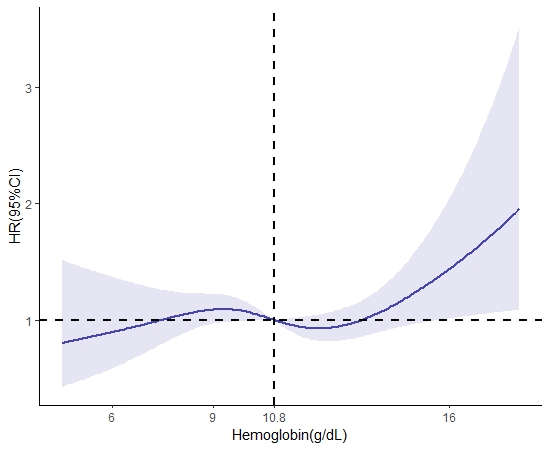


**（c）**

**（b））**

**（a）**

**Figure S3** Restricted cubic spline (RCS) curves analysis of laboratory parameters-related indicators and 7-day mortality. (a) WBC count; (b) Hemoglobin; (C) INR: international normalized ratio

**Table S1. Univariate and multivariate analyses with Cox regression on short-term mortality**

| **7-day mortality** |  | **Univariate analysis** | | **p-value** | **Multivariate analysis** | **p-value** |
| --- | --- | --- | --- | --- | --- | --- |
|  |  | **No.** | **HR (95%CI)** |  | **HR (95%CI)** |  |
| Age |  | 10368 | 1.04(1.04,1.05) | <0.001 | 1.04(1.03,1.04) | <0.001 |
| Sex | Male | 6099 | 0.98(0.83,1.16) | 0.852 |  |  |
|  | Female | 4269 |  |  |  |  |
| SpO2/FiO2 |  | 10368 | 0.99(0.99,099) | <0.001 | 0.99(0.99,099) | <0.001 |
| WBC count |  | 10368 | 1.01(1.01,1.02) | <0.001 | 1.01(1.01,1.01) | <0.001 |
| INR |  | 10368 | 1.21(1.15,1.27) | <0.001 | 1.15(1.09,1.22) | <0.001 |
| Hypertension | Yes | 5488 | 0.84(0.71,0.99) | 0.040 |  |  |
|  | No | 4880 |  |  |  |  |
| Asthma | Yes | 855 | 0.63(0.43,0.91) | 0.013 | 0.71 (0.49,1.03) | 0.073 |
|  | No | 9513 |  |  |  |  |
| COPD | Yes | 920 | 1.40(1.08,1.81) | 0.011 |  |  |
|  | No | 9448 |  |  |  |  |
| Diabetes | Yes | 3553 | 1.25(1.06,1.48) | 0.009 | 1.26(1.06,1.49) | 0.009 |
|  | No | 6815 |  |  |  |  |
| Heart failure | Yes | 3371 | 1.38(1.16,1.63) | <0.001 |  |  |
|  | No | 6997 |  |  |  |  |
| Myocardial infarction | Yes | 1259 | 2.53(2.09,3.06) | <0.001 | 2.40(1.98,2.91) | <0.001 |
|  | No | 9109 |  |  |  |  |
| Stroke | Yes | 26 | 3.02(1.13,8.08) | 0.028 | 4.82(1.80,12.91) | 0.002 |
|  | No | 10342 |  |  |  |  |
| Glucocorticoid therapy | Yes | 2970 | 1.05(0.88,1.26) | 0.596 |  |  |
|  | No | 7398 |  |  |  |  |
| Statin therapy | Yes | 5184 | 0.48(0.41,0.58) | <0.001 | 0.49(0.41,0.58) | <0.001 |
|  | No | 5184 |  |  |  |  |
| Aspirin therapy | Yes | 6026 | 0.46(0.39,0.55) | <0.001 | 0.49(0.41,0.59) | <0.001 |
|  | No | 4342 |  |  |  |  |

HR: hazard ratio; WBC: white blood cell; INR: international normalized ratio; COPD: chronic obstructive pulmonary disease

**Table S2. The demographic and clinical characteristics of ARDS patients with glucocorticoid treatment or not before and after PSM (added the cholesterol-related data)**

| **Character** | **Original cohort** | | **P-value** | **Matched cohort** | | **P-value** |
| --- | --- | --- | --- | --- | --- | --- |
|  | **Non-**  **glucocorticoid** | **Glucocorticoid** |  | **Non-**  **glucocorticoid** | **Glucocorticoid** |  |
| **Patients** | N=836 | N=369 |  | N=276 | N=276 |  |
| Age [years, M(IQR)] | 69 [59, 79] | 66 [57, 73] | <0.001 | 68 [56, 77] | 67 [60, 75] | 0.970 |
| Sex |  |  | 0.002 |  |  | 0.733 |
| Male [n (%)] | 514 (61.5) | 191 (51.8) |  | 146 (52.9) | 151 (54.7) |  |
| Female [n (%)] | 322 (38.5) | 178 (48.2) |  | 130 (47.1) | 125 (45.3) |  |
| **Vital signs** |  |  |  |  |  |  |
| Heart Rate [/min, M(IQR)] | 115 [102, 131] | 120 [105, 136] | 0.003 | 119 [105, 138] | 119 [105, 133] | 0.726 |
| Temperature [℃, M(IQR)] | 38.0 [37.5, 38.7] | 37.8 [37.3, 38.6] | 0.041 | 38.0 [37.5, 38.8] | 37.9 [37.3, 38.6] | 0.108 |
| SBP [mmHg, M(IQR)] | 169 [151, 188] | 173 [156, 188] | 0.047 | 171 [153, 191] | 172 [154, 188] | 0.896 |
| DBP [mmHg, M(IQR)] | 42 [34, 48] | 41 [33, 48] | 0.582 | 41 [33, 47] | 42 [33, 48] | 0.198 |
| RR [/min, M(IQR)] | 33 [29, 39] | 34 [29, 39] | 0.133 | 34 [29, 39] | 34 [29, 40] | 0.669 |
| Spo2 [%, M(IQR)] | 90 [85, 92] | 89 [84, 92] | 0.010 | 89 [83, 92] | 89 [85, 91] | 0.850 |
| Spo2/FiO2 [M(IQR)] | 200 [176, 233] | 208 [174, 233] | 0.636 | 203 [175, 233] | 205 [174, 230] | 0.901 |
| **Laboratory data** |  |  |  |  |  |  |
| WBC [× 10^9/L, M(IQR)] | 10.5 [7.8, 13.8] | 9.6 [6.3, 13.3] | 0.001 | 10.5 [7.6, 14.1] | 9.9 [6.7, 13.9] | 0.390 |
| Platelet [× 10^9/L, M(IQR)] | 203.0 [160.8, 259.0] | 181.00 [116.0, 263.0] | <0.001 | 206.0 [153.0, 253.0] | 190.0 [133.8, 279.5] | 0.495 |
| Hemoglobin [g/L, M(IQR)] | 12.0 [10.4, 13.5] | 11.0 [9.2, 12.8] | <0.001 | 11.5 [10.2, 12.9] | 11.5 [9.8, 13.3] | 0.981 |
| INR [, M(IQR)] | 1.2 [1.1, 1.4] | 1.2 [1.1, 1.6] | 0.002 | 1.2 [1.1, 1.4] | 1.2 [1.1, 1.4] | 0.976 |
| TC [mg/dL, M(IQR)] | 141.0 [112.0, 169.3] | 130.0 [90.0, 168.0] | 0.001 | 139.0 [109.0, 167.0] | 143.0 [104.0, 177.3] | 0.486 |
| HDL-C [mg/dL, M(IQR)] | 40.0 [31.0, 50.0] | 34.0 [23.0, 48.0] | <0.001 | 38.0 [28.0, 48.0] | 37.0 [26.0, 50.0] | 0.560 |
| Non-HDL-C [mg/dL, M(IQR)] | 99.0 [74.0, 124.3] | 91.0 [62.0, 127.0] | 0.011 | 97.0 [71.0, 122.0] | 98.0 [67.8, 131.0] | 0.447 |
| **Clinically scores** |  |  |  |  |  |  |
| CCI [M(IQR)] | 6 [5, 8] | 7 [5, 9] | 0.003 | 6 [5, 8] | 7 [5, 9] | 0.324 |
| SAPS II scores [M(IQR)] | 36 [29, 45] | 40 [32, 50] | <0.001 | 37 [30, 49] | 38 [30, 48] | 0.483 |
| SOFA scores [M(IQR)] | 1 [0, 3] | 2 [0, 5] | <0.001 | 2 [0, 4] | 1 [0, 4] | 0.594 |
| **Comorbidity** |  |  |  |  |  |  |
| Hypertension [n (%)] | 456 (54.5) | 221 (59.9) | 0.097 | 152 (55.1) | 161 (58.3) | 0.492 |
| Asthma [n (%)] | 58 (6.9) | 39 (10.6) | 0.043 | 26 (9.4) | 26 (9.4) | 1.000 |
| COPD [n (%)] | 49 (5.9) | 33 (8.9) | 0.067 | 20 (7.2) | 27 (9.8) | 0.360 |
| Diabetes [n (%)] | 277 (33.1) | 143 (38.8) | 0.069 | 104 (37.7) | 103 (37.3) | 1.000 |
| Heart failure [n (%)] | 247 (29.5) | 109 (29.5) | 1.000 | 80 (29.0) | 86 (31.2) | 0.643 |
| Myocardial infarction [n (%)] | 129 (15.4) | 51 (13.8) | 0.526 | 44 (15.9) | 42 (15.2) | 0.907 |
| Stroke [n (%)] | 10 (1.2) | 2 (0.5) | 0.460 | 3 (1.1) | 2 (0.7) | 1.000 |
| Pulmonary embolism [n (%)] | 48 (5.7) | 21 (5.7) | 1.000 | 16 (5.8) | 16 (5.8) | 1.000 |
| Venous thrombosis [n (%)] | 27 (3.2) | 22 (6.0) | 0.040 | 14 (5.1) | 17 (6.2) | 0.712 |
| Tumor [n (%)] | 5 (0.6) | 3 (0.8) | 0.969 | 1 (0.4) | 3 (1.1) | 0.616 |
| **Treatment** |  |  |  |  |  |  |
| Statin therapy [n (%)] | 491 (58.7) | 190 (51.5) | 0.023 | 155 (56.2) | 157 (56.9) | 0.932 |
| Aspirin therapy [n (%)] | 531 (63.5) | 263 (71.3) | 0.011 | 187 (67.8) | 185 (67.0) | 0.928 |
| Mechanical ventilation [n (%)] | 621 (74.3) | 291 (78.9) | 0.102 | 215 (77.9) | 208 (75.4) | 0.546 |
| **Primary-outcome** |  |  |  |  |  |  |
| 7-day mortality [n (%)] | 48 (5.7) | 10 (2.7) | 0.034 | 15 (5.4) | 10 (3.6) | 0.413 |

Continuous variables are presented as median (IQR) for non-normally distributed data. Categorical variables were expressed as the numbers and percentages (%). SBP: systolic blood pressure; DBP: diastolic blood pressure; RR: respiratory rate; Spo2: percutaneous oxygen saturation; WBC: white blood cell; INR: international normalized ratio; TC: total Cholesterol; HDL-C: High-Density Lipoprotein cholesterol; non-HDL-C: non- high-density lipoprotein cholesterol; CCI: Charlson Comorbidity Index; SAPS II score: simplified acute physiology score; SOFA score: sequential organ failure assessment score; COPD: chronic obstructive pulmonary disease

**Table S3. The demographic and clinical characteristics of ARDS patients with statin treatment or not before and after PSM (added the cholesterol-related data)**

| **Character** | **Original cohort** | | **P-value** | **Matched cohort** | | **P-value** |
| --- | --- | --- | --- | --- | --- | --- |
|  | **Non-statin** | **Statin** |  | **Non-statin** | **Statin** |  |
| **Patients** | N=524 | N=681 |  | N=401 | N=401 |  |
| Age [years, M(IQR)] | 67 [59, 79] | 68 [59, 77] | 0.324 | 68 [60, 79] | 68 [60, 76] | 0.178 |
| Sex |  |  | 0.034 |  |  | 0.775 |
| Male [n (%)] | 325 (62.0) | 380 (55.8) |  | 236 (58.9) | 231 (57.6) |  |
| Female [n (%)] | 199 (38.0) | 301 (44.2) |  | 165 (41.1) | 170 (42.4) |  |
| **Vital signs** |  |  |  |  |  |  |
| Heart Rate [/min, M(IQR)] | 115 [102, 131] | 117 [104, 132] | 0.341 | 115 [103, 131] | 117 [104, 131] | 0.987 |
| Temperature [℃, M(IQR)] | 37.9 [37.4, 38.7] | 37.9 [37.4, 38.7] | 0.506 | 38.0 [37.5, 38.7] | 37.9 [37.4, 38.7] | 0.539 |
| SBP [mmHg, M(IQR)] | 171 [155, 187] | 169 [150, 190] | 0.260 | 171 [155, 188] | 168 [150, 191] | 0.284 |
| DBP [mmHg, M(IQR)] | 42 [34, 49] | 41 [34, 48] | 0.210 | 42 [35, 49] | 42 [34, 48] | 0.418 |
| RR [/min, M(IQR)] | 33 [28, 38] | 34 [29, 39] | 0.023 | 33 [28, 39] | 33 [29, 39] | 0.851 |
| Spo2 [%, M(IQR)] | 90 [85, 92] | 89 [85, 92] | 0.826 | 90 [85, 92] | 90 [85, 92] | 0.851 |
| Spo2/FiO2 [M(IQR)] | 207 [178, 235] | 200 [174, 230] | 0.054 | 205 [178, 235] | 200 [174, 233] | 0.281 |
| **Laboratory data** |  |  |  |  |  |  |
| WBC [× 10^9/L, M(IQR)] | 10.4 [7.3, 13.7] | 9.9 [7.7, 13.5] | 0.966 | 10.7 [7.6, 13.7] | 9.9 [7.6, 13.0] | 0.166 |
| Platelet [× 10^9/L, M(IQR)] | 185.0 [126.8, 250.3] | 208.0 [163.0, 266.0] | <0.001 | 191.0 [142.0, 256.0] | 202.0 [161.0, 254.0] | 0.084 |
| Hemoglobin [g/L, M(IQR)] | 11.5 [9.9, 13.3] | 11.8 [10.1, 13.4] | 0.322 | 11.7 [10.5, 13.4] | 11.7 [10.1, 13.4] | 0.787 |
| INR [, M(IQR)] | 1.2 [1.1, 1.5] | 1.2 [1.1, 1.4] | <0.001 | 1.2 [1.1, 1.5] | 1.2 [1.1, 1.4] | 0.212 |
| TC [mg/dL, M(IQR)] | 134.5 [101.0, 167.0] | 141.0 [110.0, 171.0] | 0.003 | 142.0 [109.0, 173.0] | 140.0 [110.0, 171.0] | 0.945 |
| HDL-C [mg/dL, M(IQR)] | 36.0 [26.0, 49.0] | 39.0 [30.0, 49.0] | 0.004 | 39.0 [28.0, 51.0] | 39.0 [30.0, 49.0] | 0.607 |
| Non-HDL-C [mg/dL, M(IQR)] | 95.0 [66.0, 120.0] | 99.0 [72.0, 129.0] | 0.007 | 99.0 [71.0, 124.0] | 98.0 [72.0, 127.0] | 0.829 |
| **Clinically scores** |  |  |  |  |  |  |
| CCI [M(IQR)] | 7 [5, 9] | 6 [5, 8] | 0.041 | 6 [5, 8] | 6 [5, 8] | 0.283 |
| SAPS II scores [M(IQR)] | 38 [31, 47] | 37 [29, 46] | 0.038 | 37 [31, 46] | 36 [28, 47] | 0.388 |
| SOFA scores [M(IQR)] | 2 [0, 4] | 1 [0, 3] | 0.004 | 1 [0, 3] | 2 [0, 3] | 0.194 |
| **Comorbidity** |  |  |  |  |  |  |
| Hypertension [n (%)] | 313 (59.7) | 364 (53.5) | 0.034 | 223 (55.6) | 228 (56.9) | 0.776 |
| Asthma [n (%)] | 44 (8.4) | 53 (7.8) | 0.778 | 34 (8.5) | 31 (7.7) | 0.796 |
| COPD [n (%)] | 39 (7.4) | 43 (6.3) | 0.512 | 31 (7.7) | 24 (6.0) | 0.402 |
| Diabetes [n (%)] | 200 (38.2) | 220 (32.3) | 0.040 | 144 (35.9) | 132 (32.9) | 0.414 |
| Heart failure [n (%)] | 141 (26.9) | 215 (31.6) | 0.090 | 110 (27.4) | 117 (29.2) | 0.638 |
| Myocardial infarction [n (%)] | 79 (15.1) | 101 (14.8) | 0.971 | 67 (16.7) | 58 (14.5) | 0.436 |
| Stroke [n (%)] | 5 (1.0) | 7 (1.0) | 1.000 | 4 (1.0) | 7 (1.7) | 0.544 |
| Pulmonary embolism [n (%)] | 26 (5.0) | 43 (6.3) | 0.381 | 21 (5.2) | 28 (7.0) | 0.376 |
| Venous thrombosis [n (%)] | 20 (3.8) | 29 (4.3) | 0.812 | 13 (3.2) | 19 (4.7) | 0.367 |
| Tumor [n (%)] | 3 (0.6) | 5 (0.7) | 1.000 | 2 (0.5) | 3 (0.7) | 1.000 |
| **Treatment** |  |  |  |  |  |  |
| Glucocorticoid use [n (%)] | 179 (34.2) | 190 (27.9) | 0.023 | 116 (28.9) | 104 (25.9) | 0.384 |
| Aspirin therapy [n (%)] | 353 (67.4) | 441 (64.8) | 0.376 | 261 (65.1) | 269 (67.1) | 0.602 |
| Mechanical ventilation [n (%)] | 401 (76.5) | 511 (75.0) | 0.596 | 294 (73.3) | 299 (74.6) | 0.748 |
| **Primary-outcome** |  |  |  |  |  |  |
| 7-day mortality [n (%)] | 40 (7.6) | 18 (2.6) | <0.001 | 32 (8.0) | 11 (2.7) | 0.002 |

Continuous variables are presented as median (IQR) for non-normally distributed data. Categorical variables were expressed as the numbers and percentages (%). SBP: systolic blood pressure; DBP: diastolic blood pressure; RR: respiratory rate; Spo2: percutaneous oxygen saturation; WBC: white blood cell; INR: international normalized ratio; TC: total Cholesterol; HDL-C: High-Density Lipoprotein cholesterol; non-HDL-C: non- high-density lipoprotein cholesterol; CCI: Charlson Comorbidity Index; SAPS II score: simplified acute physiology score; SOFA score: sequential organ failure assessment score; COPD: chronic obstructive pulmonary disease
